# Prohibition in Atlanta

**Published:** 1885-11-20

**Authors:** 


### PROHIBITION IN ATLANTA.

Atlanta is now deeply agitated over the question of the Prohibition of Spirituous Liquors in the city, and the county of Fulton—which is to be decided at an election on the 25th instant.

There is a provision in the bill preventing the use of the ordinary liquors by Druggists in preparing prescriptions, but allowing the use, or substitution, of proof alcohol for medicinal purposes. This feature of the bill has led to considerable interest on the part of medical practitioners in the city. A number of those who oppose the bill on account of this feature, have expressed themselves through the newspapers, while as many, favoring the bill, have also published their views on the subject. Public meetings have been held by the physicians for and against Prohibition, and resolutions expressive of their opinions adopted. It would appear that a majority of the profession in the city favor Prohibition. The clause confining druggists and physicians to the use of proof alcohol in prescriptions has proven to be the chief stumbling block. Many who favor Prohibition will vote against the bill on this account.

As editor of a Medical journal, our opinion has been asked for on this point. Without entering upon the details of the argument touching the difference between the ordinary liquors and proof alcohol, which have been so much discussed, we simply give it as our opinion that, while the plain alcohol can not be made to substitute, satisfactorily, every form of alcoholic drink in the make-up of prescriptions, it can be so substituted for most of them ; and it is possible, in many instances where liquors are now used as medicine, to dispense with them altogether, and substitute other agents of equal efficiency.

We confess that we would like the bill much better if this provision had been omitted, as it imposes inconveniences which are unnecessary, while adding nothing to the benefits sought to be accomplished by such a law. Notwithstanding this fact, we regard the evils flowing from bar rooms and the retail of spirituous liquors so prodigious that the sentiments of the Christian world, and the moral force of the entire community, should be arrayed against the liquor traffic, and in favor of the law, despite the objection which has been mentioned.

There is one fact in connection with the use of intoxicating liquors, which thinking men in the Medical profession are beginning to regard as of grave and serious importance in its bearings upon the future health and welfare of mankind ; it is, that Drunkenness is a disease, and one that is transmitted from the drunkard to his offspring ; that it is on the increase, and, if no check is imposed to the dreadful habit of drunkenness, the time will come, and at no distant period, when the consequences to society will be appalling.

Those in the Medical profession who have devoted special attention to the study of nervous diseases have come to regard dipsomania, or alcoholic mania, as a true and well-defined disease, one of the characteristics of which is that at certain periods the proclivity for drink is such that the moral sense of the patient is overcome by the insatiable thirst for drink, and his power to resist the temptation when presented to him is wholly lost. Herein we find a controlling argument against moral suasion, and a clear explanation why this method of deliverance from the evil has heretofore failed, and why it ought no longer to be relied upon.

It is now a recognized fact that drunkenness may be, and is often, entailed upon the offspring by hereditary transmission from parent to child. The influence thus transmitted, may manifest itself in different phases of nervous perversion. In some, the trouble may show itself in a tendency to the morphine habit or to other disordered mental proclivities—not unfrequently in insanity.

What, then, is the duty of the Christian world, and, indeed, of every well-wisher to our race, in regard to this subject? The question is momentous, not only in its present but in its prospective bearing upon society, and the world. Solomon said, "The prudent man foreseeth the evil and avoideth it." The highest and best morality regards the welfare not only of ourselves but of our posterity. Prohibition, as now proposed, may not accomplish all that is desired, but it will certainly do some good. It will remove the social attractions of the saloons and bar rooms, so fascinating to the young and inexperienced. It will make the procuring of strong drink inconvenient and difficult, and thus prevent a great deal of drunkenness that would otherwise result; and it will, at least, prove a stepping-stone to other and more efficient methods, which, we trust, the increasing moral sense and Christian sentiment of the future will bring to bear for the rescue of mankind from this gigantic and appalling evil.

